# A Mobile Outdoor Augmented Reality Method Combining Deep Learning Object Detection and Spatial Relationships for Geovisualization

**DOI:** 10.3390/s17091951

**Published:** 2017-08-24

**Authors:** Jinmeng Rao, Yanjun Qiao, Fu Ren, Junxing Wang, Qingyun Du

**Affiliations:** 1 School of Resources and Environmental Science, Wuhan University, 129 Luoyu Road, Wuhan 430079, China; rokim@whu.edu.cn (J.R.); qiaoyanjun8@whu.edu.cn (Y.Q.); renfu@whu.edu.cn (F.R.); edenwjx@whu.edu.cn (J.W.); 2 Key Laboratory of GIS, Ministry of Education, Wuhan University, 129 Luoyu Road, Wuhan 430079, China; 3 Key Laboratory of Digital Mapping and Land information Application Engineering, National Administration of Surveying, Mapping and Geoinformation, Wuhan University, 129 Luoyu Road, Wuhan 430079, China; 4 Collaborative Innovation Center of Geospatial Technology, Wuhan University, 129 Luoyu Road, Wuhan 430079, China

**Keywords:** geovisualization, outdoor augmented reality, deep learning, object detection, Inertial Measurement Unit

## Abstract

The purpose of this study was to develop a robust, fast and markerless mobile augmented reality method for registration, geovisualization and interaction in uncontrolled outdoor environments. We propose a lightweight deep-learning-based object detection approach for mobile or embedded devices; the vision-based detection results of this approach are combined with spatial relationships by means of the host device’s built-in Global Positioning System receiver, Inertial Measurement Unit and magnetometer. Virtual objects generated based on geospatial information are precisely registered in the real world, and an interaction method based on touch gestures is implemented. The entire method is independent of the network to ensure robustness to poor signal conditions. A prototype system was developed and tested on the Wuhan University campus to evaluate the method and validate its results. The findings demonstrate that our method achieves a high detection accuracy, stable geovisualization results and interaction.

## 1. Introduction

Geovisualization, or geographic visualization, is an efficient way to describe the real geographic world through visual means, thereby making complex geographic data and information intuitive and easy to understand. An appropriate geovisualization method can provide prompt insight and understanding to support real-world knowledge construction and decision-making [1]. Recently, as a tool, a process and a mode of thought, geovisualization has been widely used in environmental monitoring [2], spatial decision-making [3], urban mobility [4], meteorology [5], and archaeology [6], among other fields. However, traditional geovisualization methods suffer from several drawbacks when facing various increasingly challenging representation needs. On the one hand, traditional geovisualization usually refers to 2D/3D cartographic visualization, which, to some degree, is isolated from the real world because it involves creating another “world” (such as a map or a virtual environment) to describe the real world, and this isolation may result in improper spatial cognition or may even produce incorrect information. For example, the limitation of small screens on mobile or embedded devices adds difficulty to user’s cognitive mapping [7]. On the other hand, interaction, as an important dimension of geovisualization [8], is vital to the user experience. However, the outputs of traditional geovisualization are usually limited to paper maps or electronic maps, which provide limited modes of interaction for users.

Augmented Reality (AR) is a promising branch of technology that offers new modes of visualization, navigation and user interaction [9]. In particular, outdoor AR technology provides new opportunities for visualizing geographic data and information in a more direct and intuitive way in an outdoor geographical environment. Many current AR methods and applications are based on visual fiducial markers [10,11,12]. However, such methods demand a controlled environment (usually an indoor environment) and require markers to be placed in advance. In the case of outdoor geographical environments, it is usually not practical to cover such an uncontrolled environment with markers [13]. To date, many attempts have been made to develop AR methods targeting outdoor environments, and some of them have already been applied to enhance the results of geovisualization [14,15,16]. In most outdoor AR methods and applications, a Global Positioning System (GPS) receiver, inertial sensors and magnetic sensors are generally used to obtain the relative distances and orientations of users and geographic objects; however, such sensors suffer from many problems, such as deterioration in the GPS precision and drift and distortion in the output of inertial and magnetic sensors [17], which sometimes lead to unsatisfactory results. As an essential area of AR research, vision-based natural feature detection [17] enables the extraction of object features from uncontrolled environments for classification and localization and has been widely used in outdoor AR methods for detection and tracking [18,19]. Traditional vision-based natural feature detection methods, such as natural keypoint detectors (e.g., SIFT [20], SURF [21], HOG [22], and Haar [23]) or edge-based approaches [24], can achieve high positional accuracy but are overly sensitive to motion blur, changes in lighting conditions, occlusion, and other such phenomena and have difficulty coping with multiple objects or detection at multiple scales or from multiple perspectives, which frequently results in instability and even failure. As is noted in [9], a single technology is not always sufficient for registration and interaction in AR; therefore, it is necessary to integrate various technologies together.

The purpose of the study reported in this paper was to develop a robust, fast and markerless outdoor AR method for execution on mobile or embedded devices in uncontrolled outdoor environments to achieve registration, geovisualization and interaction that can adapt to various challenging outdoor conditions, such as motion blur, rotation, occlusion, and multiple objects, scales and perspectives. To achieve this goal, a lightweight, energy-efficient but powerful vision-based geographic object detection approach for outdoor mobile AR is needed, and the vision-based detection results for geographic objects should be combined with their corresponding spatial relationships to achieve the precise registration of virtual objects, with the help of the host device’s built-in GPS receiver, Inertial Measurement Unit (IMU) and magnetometer, to serve as the basis for subsequent AR geovisualization and interaction. Moreover, for robustness against the poor signal conditions found in many challenging outdoor environments, the method should be sufficiently flexible and independent; achieving this goal requires a small model size and eliminating any dependence on the network to the greatest possible extent. Our method can accurately detect geographic objects in near real time with sufficient robustness and can then augment them by registering and visualizing virtual objects generated based on geospatial information. Our method provides a new AR-based means of geovisualization and interaction to assist users in understanding and interacting with the geographical environment in an intuitive manner, thereby enriching the user experience, which is expected to be beneficial in many diverse applications, such as urban planning, environmental monitoring and spatial decision-making.

The remainder of this paper is organized as follows: in Section 2, the development of outdoor AR systems is introduced, and recent deep-learning-based object detection methods are reviewed. In Section 3, a lightweight vision-based deep learning object detection approach for outdoor AR on mobile or embedded devices is proposed and evaluated. In Section 4, we describe our proposed method of mobile outdoor AR for registration, geovisualization and interaction, which combines vision-based detection results and spatial relationships with the help of the host device’s built-in GPS receiver, IMU and magnetometer. In Section 5, a prototype system we developed using the proposed method is presented, which was tested on the Wuhan University campus to evaluate the method and validate its results, and a discussion of the findings is presented. Conclusions and future work are outlined in Section 6.

## 2. Related Work

### 2.1. Outdoor Augmented Reality

Due to the huge range of uncontrolled outdoor environments, a combination of multiple sensors is required in an outdoor AR system to accomplish the registration process, such as a GPS receiver for locations and distances, inertial sensors and magnetic sensors for orientations [25,26,27,28,29,30,31]. Many early outdoor AR systems and applications, represented by [26], use wearable computer systems (e.g., Head Mount Display devices) to access information. Those works show potential applications of outdoor AR but have large registration errors. Reference [30] presents an outdoor AR system which uses rate gyros, compass and tilt orientation sensor to achieve accurate motion-stabilized registration, while this system assumes that the real-world objects are distant (e.g., 500 + meters), and it is only operated and tested at static locations.

Due to the serious systematic errors produced by the sensors, many outdoor AR systems currently employ computer vision technologies for achieving precise registration [32,33,34]. Some methods utilize vision tracking technologies to reduce the drift of inertial sensors. Reference [33] proposes an early hybrid AR system where an inertial system provides frame-to-frame camera orientation predictions to increase the robustness and computing efficiency, and a vision system corrects the accumulated drift of the inertial system. Reference [34] presents a real-time gyros-vision hybrid tracking system which uses line-based vision tracking technology to detect and match line features on buildings that occur in outdoor environments and to stabilize the gyro drift. Those systems are overly sensitive to the rapid viewpoint displacement and rotation and run on a workstation or a PC, which limits their mobility. Reference [35] introduces mobile infrared beacons which are added to outdoor environments as the references to correct errors in inertial sensors and GPS receiver, while this method requires setting beacons on vehicles, persons or static locations in advance, and the paper only shows a simulation experiment. There are also many methods which use model-based tracking technologies to achieve precise registration for outdoor AR [36,37,38], but those methods rely heavily on Computer Aided Design (CAD) models of buildings.

Another stream of research focuses on vision-based natural feature detection for object recognition or localization in the context of AR. Reference [39] describes an early AR system based on SIFT for object localization, while the system runs on a laptop and is only tested in indoor environments. Reference [40] demonstrates a streaming mobile AR system using SURF features for recognition and tracking, but the main computing tasks such as feature extracting and matching are implemented on a server, and the network(e.g., 3G network) latency between mobile phones and the server accounts for 31% of total time cost. In [41], the SURF algorithm is implemented on a mobile phone for outdoor AR. The AR techniques presented in [42] use modified SIFT and Ferns to achieve real-time detection and tracking on mobile phones. Reference [43] presents a markerless AR detection approach using a Random Forest classifier for interesting point matching in uncontrolled environments. Reference [44] combines cloud-offloaded computer vision with a location-free geometric representation to prune down the visual search space for reducing the latency in mobile AR applications. In summary, most of those methods either are not very robust to various visual conditions or have difficulty coping with multiple objects, multiple scales or multiple perspectives phenomena. Some of them are based on the client/server architecture, resulting in network latency and vulnerability to poor signal conditions.

Compared with the aforementioned researches, our method utilizes a deep-learning-based object detection approach which can adapt to various challenging outdoor visual conditions with sufficient robustness, and the approach is lightweight and energy-efficient enough to be completely implemented on low-power mobile or embedded devices, eliminating the dependence on the network and eradicating latency. Besides, our method considers spatial relationships, which can refine the vision-based detection results of geographic objects and is able to correctly distinguish between geographic objects with similar visual properties.

### 2.2. Deep-Learning-Based Object Detection

Object detection is currently attracting considerable attention in many fields, such as AR [45], autonomous driving [46], remote sensing [47,48], ecology investigation [49], and medical science [50]. There are many commonly used object detection datasets, such as the ImageNet [51], PASCAL VOC [52], and Microsoft COCO [53] datasets. Over the past few decades, many object detection methods have continuously emerged, and some of them demonstrate state-of-the-art performance. Traditional object detection methods, however, have several drawbacks. First, the performance of traditional methods strongly depends on the design of the feature extractors, which requires careful engineering and domain expertise [54], and even still, these manually crafted features are not very robust to various visual phenomena, such as lighting changes, motion blur and different perspectives or scales. Second, traditional region selection approaches are mainly based on sliding window methods, which have a high time complexity and produce a large number of redundant windows, leading to a massive number of useless computations.

Since Krizhevsky et al. [55] won the classification task of ILSVRC 2012 with the lowest top-5 error rate (15.3%, much lower than that of the traditional machine learning method SIFT+FVs [56], which was 26.2%) using a large deep Convolutional Neural Network (CNN) named AlexNet, the heyday of deep learning methods in computer vision has arrived. Instead of relying on manual feature engineering, deep learning methods automatically discover from raw data the representations needed for classification or detection, thereby taking advantage of the increasing availability of computational resources and data [54]. It turns out that features extracted from a trained deep CNN can be repurposed for many computer vision tasks, making a CNN an efficient “black box feature extractor”. OverFeat [57] is an early object detection method using a CNN with a multi-scale sliding window approach for classification, localization and detection, which won the Classification + Localization task of ILSVRC 2013.

Compared with the traditional sliding window algorithm, the region proposal method (also called the detection proposal method) [58] is a better solution for region selection. The region proposal method assumes that all objects in an image share common visual properties that distinguish them from the background, which allows us to develop a method for identifying region proposals, which are the candidate regions in an image that are more likely to contain objects. Several region proposal approaches, such as selective search [59] and objectness [60], are widely used in deep learning methods to reduce the number of regions and the time complexity while maintaining high object recalls.

**Figure 1:**
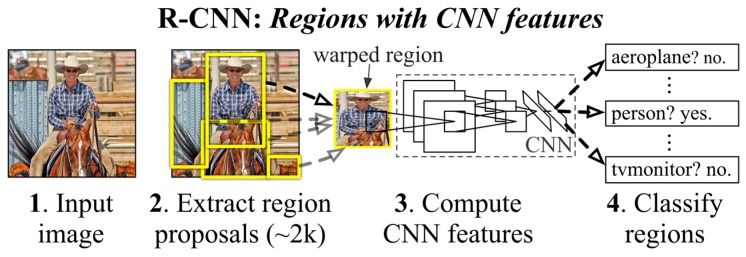
An overview of R-CNN (from [61]). This method first takes an image as an input, then extracts approximately 2000 region proposals and computes features from each proposed region using a deep CNN, and finally uses linear SVMs to classify those proposed regions.

Recently, many deep learning methods have been proposed that combine the region proposal method with CNNs for object detection to achieve significant performance. R-CNN [61] (an overview is shown in Figure 1) combines selective search with a CNN to obtain 2000 region proposals and extract features from each proposed region for detection and then applies a linear regression model to obtain bounding boxes with reduced localization errors, thereby achieving a considerable improvement over OverFeat on the ILSVRC 2013 detection dataset (increasing the mAP from 24.3% to 31.4%). SSP-Net [62] speeds up R-CNN by extracting features from the entire image only once with the help of a spatial pyramid pooling layer. Fast R-CNN [63] uses an ROI pooling layer (a simplified spatial pyramid pooling layer) and multi-task loss to improve training and testing speed as well as detection accuracy. Faster R-CNN [64] replaces the selective search process with an RPN (Region Proposal Network) for region proposal generation, thereby combining region proposal, classification and localization regression to improve speed and accuracy.

**Figure 2:**
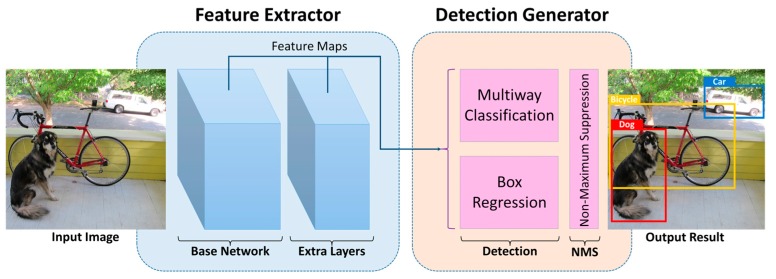
An overview of SSD. SSD first takes an image as input, then extracts features by means of a base network (e.g., a truncated VGG-16 [67] network without classification layers) and several additional feature layers to obtain multi-scale feature maps, subsequently obtains initial detection results through multiway classification and box regression using a set of convolutional filters, and finally applies Non-Maximum Suppression (NMS) to eliminate redundant results.

The region-proposal-based deep learning methods represented by the variants of R-CNN are accurate but still too slow to achieve real-time detection. Another group of methods skips the time-consuming region selection step by directly predicting confidences for classification and bounding boxes for localization, thereby dramatically enhancing the speed of detection. One of these methods is You Only Look Once (YOLO) [65], which achieves real-time performance by reframing object detection as a single regression problem, computing a global feature map and using a fully connected layer to predict both confidences and bounding boxes. Another method, Single Shot Detector (SSD, an overview is shown in Figure 2) [66], achieves faster and significantly more accurate performance (74.3% mAP at 59 FPS on an NVIDIA Titan X GPU for 300 × 300 pixel input) compared with YOLO by adding layers of feature maps at each scale and using a convolutional filter for multi-scale detection with default boxes of different sizes and ratios.

## 3. A Lightweight SSD for Mobile Outdoor AR

We choose SSD as the visual object detection approach for our mobile outdoor AR method by virtue of its high detection accuracy and speed. However, because of its enormous computational cost, it is still too slow and computationally expensive to run the original SSD without a powerful GPU, let alone on low-power mobile or embedded devices. Moreover, the heavy and complex architecture of the original SSD requires a weight file of more than 100 MB, which is excessively large for a mobile application when the storage space is limited or a large number of models are needed. Currently, a common solution is to first implement SSD on a powerful server and then allow mobile or embedded devices to send input images to the server and receive output visual detection results from the server through the network. However, this solution will inevitably lead to dependence on the network, resulting in latency and vulnerability to poor signal conditions, which should be avoided to the greatest possible extent in outdoor AR.

In brief, a local, energy-efficient and lightweight object detection method that can run on mobile devices is more suitable than one running on a server for handling various outdoor environments under poor signal conditions. Therefore, we propose lightweight SSD, a version of SSD that has been modified by changing the original SSD architecture to make it sufficiently lightweight for mobile or embedded devices. We greatly reduce the computing cost (and also the size of the weight file) to achieve near-real-time performance on mobile or embedded devices while maintaining a high detection accuracy.

### 3.1. Network Architecture

**Figure 3:**
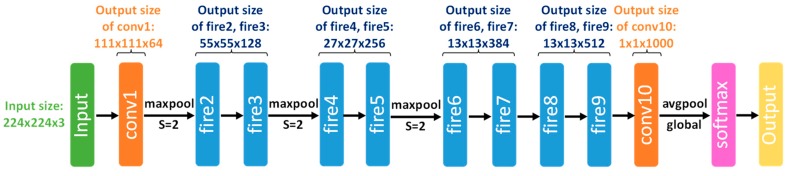
Macro architectural view of SqueezeNet v1.1 (inspired by Figure 2 in [68]). Processing begins with a convolutional layer (conv1), followed by 8 fire modules (structures proposed in [68], which have fewer parameters than normal convolutional layers without sacrificing competitive accuracy), and ends with a convolutional layer (conv10) and a softmax classifier. SqueezeNet takes as input a 224 × 224 pixel image with 3 colour channels (R, G and B).

A large proportion of the computational cost of SSD is due to the base network (e.g., truncated VGG-16 in the original SSD) and additional feature layers, which are mainly used to extract multi-scale features from the input image. Thus, we replace the heavy base network with a more lightweight one and modify the subsequent additional feature layers. SqueezeNet [68] is a very lightweight classification CNN architecture that achieves AlexNet-level accuracy on ImageNet with 50 × fewer parameters and a weight file of only 4.8 MB in size. The latest version, SqueezeNet v1.1 (the architecture is shown in Figure 3), requires 2.4 × fewer computations than the original, without sacrificing accuracy.

**Figure 4:**
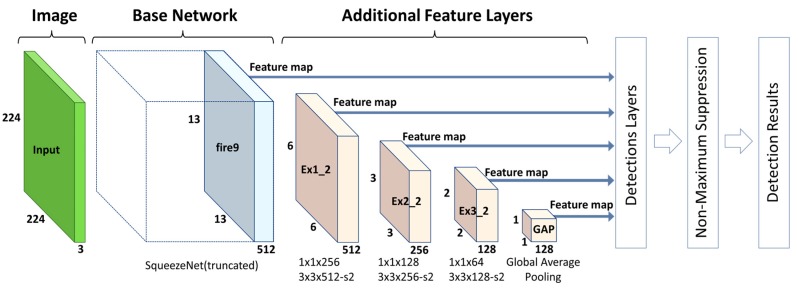
The proposed lightweight SSD architecture (inspired by Figure 2 in [66]). This architecture follows a design similar to that of the original SSD. The main differences are that it takes a 224 × 224 pixel image as input and then uses a truncated SqueezeNet (rather than VGG-16) and a series of additional layers (at lower depths than the original) to extract features from the image. The features it uses for detection are selected from 5 layers: fire9 (the last fire module in the SqueezeNet), Ex1_2, Ex2_2, Ex3_2 (three convolutional layers) and GAP (a global average pooling layer).

Therefore, to significantly reduce the computational cost of the proposed lightweight SSD approach, we use a truncated SqueezeNet architecture (with conv10 and the softmax classifier removed) as the base network and append several additional feature layers (at lower depths than the original) with decaying spatial resolution. Furthermore, whereas the original SSD takes input of 300 × 300 pixels in size and selects six layers from among both the base network layers and the additional feature layers to extract multi-scale features, we use only 224 × 224 pixel input and select only five layers. All of these modifications allow the method as a whole to achieve energy-efficient and near-real-time performance when running on mobile or embedded devices. The details of the architecture are shown in Figure 4.

### 3.2. Network Performance

As was done for the original SSD, we trained the proposed lightweight SSD on the PASCAL VOC2007 + VOC2012 trainval datasets and then tested it on the PASCAL VOC2007 test dataset to evaluate its performance. We characterize the training stage in terms of accuracy and loss values. The accuracy is the overall accuracy, including foreground object predictions and background object predictions, and the loss is the overall objective loss function used in [66], which is a weighted sum of the localization loss and the confidence loss and is defined in Equation (1):

(1) $$L=\frac{1}{N}\left(L_{conf}+\alpha L_{loc}\right)\;,$$

where N is the number of default boxes matched to any ground-truth boxes with a Jaccard overlap higher than a specific threshold (e.g., 0.5) for training. The loss is set to 0 when N is 0. $L_{conf}$ is the confidence loss, which is the softmax loss over the confidences of multiple classes. $L_{loc}$ is the localization loss, which is a Smooth L1 [63] loss between the predicted box parameters and the ground-truth box parameters. α is a weight term, which is set to 1 through cross validation. $L_{conf}$ and $L_{loc}$ are defined in Equations (2) and (3), respectively:

(2) $$L_{conf}\left(x,c\right)=-\overset{N}{\underset{i\in Pos}{\sum }}x_{ij}^{p}\log\left({\hat{c}}_{i}^{p}\right)-\underset{i\in Neg}{\sum }\log\left({\hat{c}}_{i}^{0}\right),\;{\hat{c}}_{i}^{p}=\frac{\exp\left(c_{i}^{p}\right)}{\sum_{p}\exp\left(c_{i}^{p}\right)}\;,$$

(3) $$L_{loc}\left(x,l,\;g\right)=\overset{N}{\underset{i\in Pos}{\sum }}\underset{m\in \left\{cx,\;cy,\;w,\;h\right\}}{\sum }x_{ij}^{p}{smooth}_{L1}\left(l_{i}^{m}-{\hat{g}}_{j}^{m}\right)\;,$$

where $x_{ij}^{p}=\left\{0,\;1\right\}$ is an indicator for the matching of the *i*-th default box to the *j*-th ground-truth box for category p. $c_{i}^{p}$ is the confidence of category p corresponding to the i-th default box. $l_{i}^{m}$ is one of the parameters m of the *i*-th predicted box, and ${\hat{g}}_{j}^{m}$ is one of the parameters m of the *j*-th ground-truth box, where m can be one of the central coordinates cx and cy or the width w or the height h of the default bounding box for regression, as presented in [64]. According to the hard negative mining strategy [61], the ratio between the number of positive examples Pos and the number of negative examples Neg should be 1:3 to ensure fast and stable training.

**Figure 5:**
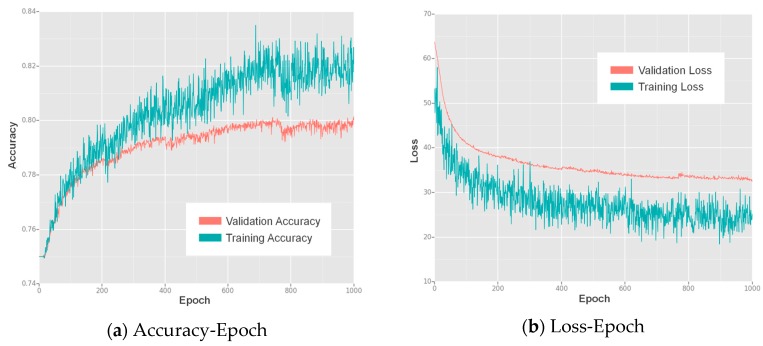
The accuracy and loss values for each epoch of training of the proposed lightweight SSD: (**a**) the accuracy-epoch curves; (**b**) the loss-epoch curves.

We trained our lightweight SSD for 1000 epochs, and the resulting accuracy-epoch and loss-epoch curves are shown in Figure 5. The SSD showed increasing accuracy and decreasing loss during training and finally reached convergence at approximately the 1000th epoch, where both the accuracy and loss values became stable.

After the training stage, we obtained a trained model with a weight file of only 17.8 MB in size, which is much smaller than that of the original and very suitable for mobile or embedded devices and applications. We then evaluated the trained lightweight SSD on the PASCAL VOC2007 test dataset to determine its mAP. Finally, we compared our approach with two popular object detection approaches: the original SSD and Fast YOLO (the fast version of YOLO with a simplified architecture). The details of this comparison are shown in Table 1.

As shown in Table 1, our approach has a lower mAP than that of the original SSD because of its less accurate base network, fewer feature layers and smaller input size; however, its mAP value is still higher than that of the other fast object detection approach, Fast YOLO, by 1%. By virtue of its lightweight architecture, the size of its weight file is only 17.8 MB, which is approximately 17% of the size of the original SSD weight file and approximately 27% of that of the Fast YOLO weight file. Regarding speed, our approach runs at 66.7 FPS on an NVIDIA GTX 1060 GPU, almost 5 times faster than the original SSD and approximately 2 times faster than Fast YOLO. On an Intel^®^ Core™ i7-6700K CPU, our approach runs at 9.1 FPS, still faster than the others. On the mobile phone, we tested only our approach and the original SSD because we did not implement a mobile version of Fast YOLO. Our framework runs at approximately 2 FPS on a Qualcomm Snapdragon 821 mobile CPU, 10 times faster than the original SSD. In summary, compared with the other two object detection approaches, our approach has the fastest speed and the smallest model size while maintaining a competitive accuracy.

## 4. A Mobile Outdoor AR Method for Geovisualization

We propose a mobile outdoor AR method for geovisualization that integrates the vision-based detection results for geographic objects obtained using the proposed lightweight SSD with their corresponding spatial relationships with the help of a GPS receiver, an IMU and a magnetometer. Important functionalities such as registration, visualization through superimposition of virtual objects and interaction are realized.

### 4.1. Overview of the Method

**Figure 6:**
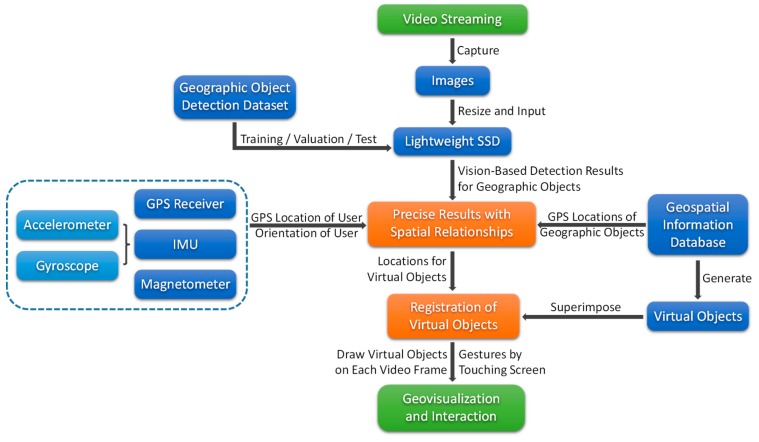
Overview of the proposed mobile outdoor AR method.

An overview of the proposed mobile outdoor AR method is shown in Figure 6. Overall, the proposed method can be divided into three important phases:

1. Training and detection with the lightweight SSD.
2. Combination of vision-based detection results and spatial relationships.
3. Registration, geovisualization and interaction.

The entire framework of the method is designed for outdoor AR on mobile or embedded devices without reliance on the network. Consequently, it takes full advantage of mobile computing capabilities and can adapt to various outdoor environments with poor signal conditions.

### 4.2. Training and Detection with the Lightweight SSD

To apply the lightweight SSD in the proposed AR method for geographic object detection, we first need to train it on a suitable geographic object detection dataset. A geographic object detection dataset should consist of a large number of images of geographic objects and their corresponding annotations, including classification labels and bounding box coordinates on the images. After appropriate training parameters have been set, such as the learning rate and weight decay, the lightweight SSD should reach convergence after training (e.g., after 1000 epochs), meaning that it has achieved a stable accuracy and is ready to be used for geographic object detection.

Afterwards, we can use this trained lightweight SSD to detect geographic objects. From the video stream generated by the visual sensor of a mobile or embedded device, we continuously and instantaneously capture frames to serve as input images. Before detection, all images need to be resized to 224 × 224 pixels because of the input size requirement of the lightweight SSD, which is almost the only necessary preprocessing step. Then, the lightweight SSD takes those images as inputs for visual detection and returns a set of results containing the information on the detected geographic objects, such as their classifications and their bounding box coordinates. Finally, the bounding box coordinates on the 224 × 224 pixel images are stretched to match the screen-size video frames (e.g., 1920 × 1080 pixels) for further registration usage.

### 4.3. Combination of Vision-Based Detection Results and Spatial Relationships

**Figure 7:**
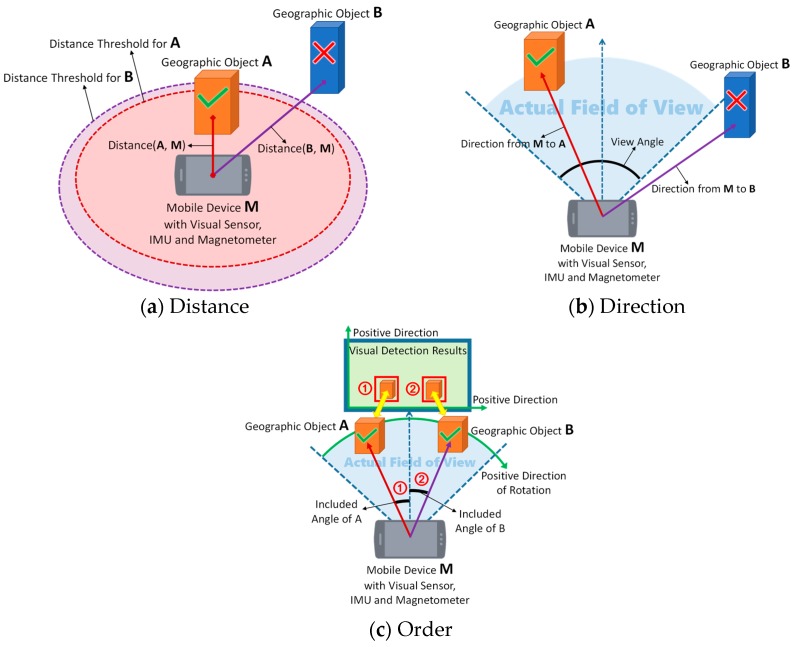
Three examples of the combination of vision-based detection results with the corresponding spatial relationships with the help of a mobile or embedded device’s built-in GPS receiver, IMU and magnetometer: (**a**) using distance thresholds to eliminate geographic object B from the vision-based detection results; (**b**) using horizontal directions and the actual horizontal field of view to eliminate geographic object B from the vision-based detection results; (**c**) matching included angles to the vision-based detection results to distinguish geographic objects A and B, which share similar visual properties.

Lightweight SSD is a purely vision-based approach and therefore does not consider spatial relationships, which are vital to the validity of vision-based detection results when applied to the real geographic world. A vision-based object detection approach has no concern for the distances and directions from the visual sensor to the objects; consequently, it may commit certain errors, such as identifying some geographic objects that are either too far away or in completely incorrect directions such that they cannot actually appear in the image at all. Even when the distances and directions are appropriate, some difficulties may still arise in obtaining precise results. For example, it is common for multiple buildings in the same housing estate to share nearly identical visual properties. An efficient object detection approach can detect these buildings with state-of-the-art performance but usually fails to distinguish individual specific buildings. Thus, it is necessary to combine vision-based detection results for geographic objects with their spatial relationships with the help of a GPS receiver, an IMU and a magnetometer to achieve the subsequent precise registration of virtual objects. In our method, we consider three aspects of the problem (examples are shown in Figure 7):

#### 4.3.1. Distance

We consider the concept of a distance threshold, which represents the maximum valid distance between the visual sensor and a geographic object. When the vision-based detection results are generated, GPS locations are queried to calculate the distance from each object to the visual sensor. The distance calculated from a pair of GPS locations is defined in Equation (4):

(4) $$\mathrm{distance}=2\arcsin\sqrt{{\left(\sin\frac{{Lat}_{a}-{Lat}_{b}}{2}\right)}^{2}+\cos\left({Lat}_{a}\right)\times \cos\left({Lat}_{b}\right)\times {\left(\sin\frac{{Lon}_{a}-{Lon}_{b}}{2}\right)}^{2}}\times R,$$

where ${Lat}_{a}$ and ${Lon}_{a}$ are the latitude and longitude of GPS location A, ${Lat}_{b}$ and ${Lon}_{b}$ are the latitude and longitude of GPS location B, and R is the radius of the Earth (km). The result is the distance (km) between locations A and B. The distances thus computed are compared with their corresponding distance thresholds, and any geographic object that does not satisfy the requirement will be discarded. An example is shown in Figure 7a.

#### 4.3.2. Direction

We also calculate the direction from the visual sensor to each geographic object using GPS locations. The direction is calculated as defined in Equation (5):

(5) $$\mathrm{direction}=\arctan\left(\frac{\cos\left({Lat}_{a}\right)\times \sin\left({Lat}_{b}\right)-\sin\left({Lat}_{a}\right)\times \cos\left({Lat}_{b}\right)\times \cos\left({Lon}_{b}-{Lon}_{a}\right)}{\sin\left({Lon}_{b}-{Lon}_{a}\right)\times \cos\left({Lat}_{b}\right)}\right)\times \frac{180}{\pi },$$

where ${Lat}_{a}$ and ${Lon}_{a}$ are the latitude and longitude of GPS location A, ${Lat}_{b}$ and ${Lon}_{b}$ are the latitude and longitude of GPS location B, and π is the ratio between the circumference of a circle and its diameter. The result is the azimuth of location B relative to location A, which represents the direction from A to B.

Then, we determine the actual horizontal field of view of the image from the horizontal view angle of the visual sensor and the information on the pose of the mobile or embedded device acquired from the IMU and magnetometer. All geographic objects whose directions lie within the actual field of view will be reserved, and the others will be eliminated from the vision-based detection results. An example is shown in Figure 7b.

#### 4.3.3. Order

To distinguish similar geographic objects with nearly identical visual properties, we determine the order of those similar objects based on their GPS locations. First, we determine the orientation of the visual sensor using the IMU and magnetometer in the mobile or embedded device. Then, we calculate the included angles between the directions from the visual sensor to each geographic object and the orientation of the visual sensor. Finally, we sort those similar geographic objects in ascending order of their included angles (i.e., from negative to positive, where the positive direction of rotation is considered to be clockwise) and then associate those geographic objects with their vision-based detection results in the image in order (e.g., from left to right). In this way, similar geographic objects can be correctly distinguished. An example is shown in Figure 7c.

It is notable that there have been many works that focus on learning spatial relationships, using spatial relationships for object recognition or refining object detection results [69,70,71,72,73]. Reference [69] presents a probabilistic model which uses the joint statistics of local appearance and position on objects for face recognition. This method achieves high detection rate on face detection while it focuses on the spatial arrangement of the features of the objects rather than the spatial relationships between the objects. Reference [70] introduces the 3D Geometric Phrase (3DGP) model that learns and reasons spatial relationships between the objects in the same 3D spatial configuration, thereby obtaining an accurate scene composition. Reference [71] describes a method using the proposed face-centric geometric descriptors and an unsupervised learning algorithm for learning object-to-object spatial relationships. However, these two methods are designed for indoor scenes and are not suitable for outdoor scenes because it is not practical to annotate objects by, for example, oriented rectangular bounding volumes [71] in an outdoor unprepared environment. Also, an additional training stage is required and a training dataset is needed in these methods. In [72], a framework is provided using a single image to model the interdependence of objects, surface orientations and camera viewpoint simultaneously in the context of the 3D scene, while one assumption of this framework is that all objects rest on the same ground plane, which is not true in many outdoor environments. Besides, this framework requires the estimation of the viewpoint (involving the horizon position and the camera height), which means the framework may not work well when the ground plane is not visible. Reference [73] presents a coherent framework with three modules for jointly detecting objects, estimating the scene layout and segmenting the supporting surfaces, thereby capturing the contextual geometrical relationship to refine the results. However, one necessary condition for this framework is that at least three objects coexist in the same image for estimating the layout.

Compared with the aforementioned methods, our method uses a simpler but still effective way which can be easily implemented on low-power mobile or embedded devices to refine the object detection results in unprepared outdoor environments. One of the advantages of our method is that there is no additional training stage (except for the training for object detection) required for generating spatial relationships, thereby allowing instantaneous calculation or modification for the spatial relationships just with the help of the sensors and the geospatial information database; Also, our method has few assumptions or prerequisites, making it a general solution to various outdoor conditions; Moreover, our method helps to correctly distinguish individual specific objects with nearly identical visual properties, which is rarely considered in other methods.

After this combination procedure, the vision-based detection results will be integrated with the corresponding spatial relationships and can be further distinguished by their order, and any that do not satisfy the specified distance and direction requirements will be discarded. Finally, with the help of a GPS receiver, an IMU and a magnetometer, we obtain a more authentic set of geographic object detection results that includes spatial relationships for registration, geospatial information visualization and interaction.

### 4.4. Registration, Geovisualization and Interaction

An authoritative definition of AR, proposed by Azuma et al. [13], is that an AR system supplements the real world with virtual (computer-generated) objects that appear to coexist in the same space as the real world. This means that the purpose of AR is not to replace the real world but to enhance it. Thus, we use a geospatial information database prepared in advance to generate virtual objects that contain geographic data and information, and these virtual objects are registered with respect to their corresponding geographic objects in the real world in accordance with their GPS locations and detected bounding box coordinates. In fact, they are also integrated with the spatial relationships of those objects because they coexist with the geographic objects in the same locations in reality.

**Figure 8:**
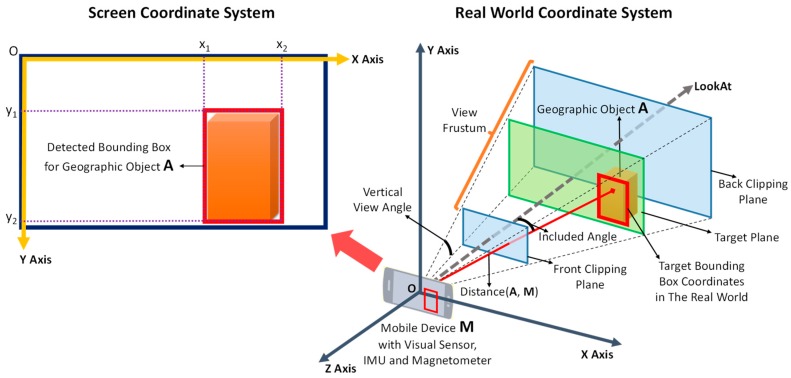
The 2D screen coordinate system, the 3D real world coordinate system and the relationships between them. A detected bounding box is described in the 2D screen coordinate system. The 3D real world coordinate system is established on the basis of the view frustum created by the visual sensor, with the origin at the centre of the visual sensor. The X and Y axes are parallel to the screen. The Z axis, which corresponds to the negative direction of the visual sensor’s orientation, is perpendicular to the screen. The 2D coordinates of the detected bounding box can be converted into target bounding box coordinates on the target plane in the 3D real world coordinate system for virtual object registration.

To enable the registration of virtual objects, the location of the geographic object in the 3D coordinate system of the real world must first be determined. Theoretically, if the size of the geographic object is known, we are able to determine the location of the geographic object in the 3D real world coordinate system just with the help of the GPS location and device posture information inferred by sensors. However, especially in the outdoor uncontrolled environment, the devices usually suffer from the deterioration in the GPS precision and the drift and distortion in the output of sensors, resulting in serious visual position deviation between the geographic object and registered virtual objects on the screen. Thus, the conversion of the coordinates of the detected bounding boxes from the 2D screen coordinate system into the 3D coordinate system of the real world is required to avoid the visual position deviation and achieve accurate registration. These two coordinate systems and their relationships are illustrated in Figure 8.

A bounding box in the vision-based detection results is defined by four pairs of coordinates, ($x_{1}$, $y_{1}$), ($x_{1}$, $y_{2}$), ($x_{2}$, $y_{1}$) and ($x_{2}$, $y_{2}$), in the 2D screen coordinate system, whose origin is at the top left of the screen. By contrast, the origin of the 3D real world coordinate system lies at the centre of the visual sensor, which can be assumed to coincide with the centre of the screen; therefore, the coordinates of the bounding box must first be converted into the screen-centred coordinate system, whose origin is at the centre of the screen. The conversion formula is given in Equation (6):

(6) $$\{\begin{array}{c} X_{c}=x-\frac{Width_{s}}{2}\\ Y_{c}=\frac{Height_{s}}{2}-y \end{array}\;,$$

where $X_{c}$ and $Y_{c}$ are the coordinates of the bounding box in the screen-centred coordinate system, x and y are the coordinates of the bounding box in the original screen coordinate system, and ${Width}_{s}$ and ${Height}_{s}$ are the width and height of the screen.

Then, these coordinates must be converted into target bounding box coordinates on the target plane in the view frustum, which is defined by two clipping planes in the 3D real world coordinate system. The target plane is perpendicular to the LookAt direction (the orientation of the visual sensor as determined by the IMU and magnetometer; also the negative direction of the *z* axis); therefore, the *z* coordinate value of the target plane is defined as shown in Equation (7):

(7) $$Z_{r}=D\times \cos\left(\theta \right)\;,$$

where D is the distance between the geographic object and the coordinate origin as calculated from the GPS locations, θ is the included angle between the direction from the coordinate origin to the geographic object and the LookAt direction, and the result $Z_{r}$ is the Z coordinate value of both the target plane and the target bounding box.

When the height and width of the target plane and the screen are properly matched, the *x* and *y* coordinates of the detected bounding box can be easily converted from the screen coordinates to the target plane coordinates. The conversion ratio is defined in Equation (8):

(8) $$\mathrm{ratio}=\frac{{Height}_{s}}{2\times \tan\left(\frac{\beta }{2}\right)\times Z_{r}}\;,$$

where ${Height}_{s}$ is the height of the screen; β is the vertical view angle of the visual sensor; and $Z_{r}$, obtained from Equation (7), is the Z coordinate value of the target plane in the real world and is also the distance between the origin of the coordinate system and the target plane. Therefore, the x and y coordinates of the target bounding box are defined as shown in Equation (9):

(9) $$\{\begin{array}{c} X_{r}=\frac{X_{c}}{ratio}\\ Y_{r}=\frac{Y_{c}}{ratio} \end{array},$$

where $X_{r}$ and $Y_{r}$ are the coordinates of the target bounding box in the 3D real world coordinate system, $X_{c}$ and $Y_{c}$ are the coordinates of the detected bounding box in the screen-centred coordinate system, and ratio is the conversion ratio from the screen coordinates to the target plane coordinates.

In this way, every pair of coordinates (x, y) of the bounding box in the 2D screen coordinate system is converted into the corresponding coordinates ($X_{r},\;Y_{r},\;Z_{r}$) of the target bounding box in the 3D real world coordinate system. Then, registration can be achieved by selecting locations near the target bounding box for the placement of virtual objects, thereby allowing those virtual objects to coexist with the detected geographic object in the same place in the real world.

After registration, these virtual objects are instantaneously superimposed in accordance with their registration locations, and all virtual objects in the view frustum are projected and superposed on every image frame of the video stream. Because the information available for the virtual objects includes their spatial relationships, objects at closer distances will be automatically placed in front of farther objects on the screen, thereby avoiding potential problems with overlay order among the virtual objects.

With regard to interaction, because of the size limitations of the screens of mobile or embedded devices, it is nearly impossible to visualize all of the available geospatial information for these small virtual objects at once; doing so would be both unnecessary and overly crowded. Therefore, we have designed an interactive way to allow users to interact with their devices using our mobile outdoor AR method by touching the screen to acquire more information or even to request additional geospatial services. Initially, all of the detected geographic objects are enhanced only with virtual labels indicating their names. When the user touches a geographic object on the screen, several additional corresponding virtual objects will fade in and present some concise geospatial information. More detailed geographic data and information can be accessed and visualized by touching these virtual objects. Other extensions, such as editing, querying and spatial analysis of geographic data and information, can also be easily executed through this mode of interaction.

## 5. Application, Validation and Discussion

We developed a prototype system using our proposed method on the Android platform. The functional modules, the geographic object detection dataset, the geospatial information database and the virtual objects were designed and built, and a performance optimization scheme targeted at mobile or embedded devices was implemented to speed up the image capture process of the visual sensor. We tested the prototype system on the Wuhan University campus to evaluate the proposed mobile outdoor AR method and validate its results. In this section, the prototype development and experiments are reported, and then a discussion is presented.

### 5.1. Prototype System Development

Our AR system was developed using Android Studio v2.2 on the Ubuntu 14.04 LTS desktop OS. Our proposed lightweight SSD approach was implemented using MXNet [74] v0.9.0, an efficient machine learning and deep learning library. We compiled the entire MXNet library into a single dynamic link library file (*.so) through the Android amalgamation method to enable its use on Android mobile or embedded devices for object detection with the help of Java Native Interface (JNI) technology. The geospatial information database for mobile or embedded devices was established using SQLite, a popular native lightweight database engine. We used OpenGL ES v2.0, a powerful graphics library, to create visual elements such as labels, virtual objects, and animation effects. The interaction functionalities were designed and implemented using Android Gestures APIs, which can recognize users’ gestures on a screen and return responses.

We optimized the image capture process for mobile or embedded devices to speed up performance. The raw data of the frames captured from the video streams produced by the visual sensors of most mobile or embedded devices are YUV data, and the default format is YCbCr_420_SP (NV21). These raw data need to be converted into 3-channel RGB data before they can be used as inputs to the lightweight SSD for detection. However, YUV-to-RGB conversion algorithms are commonly run on the CPU by default and consequently incur a high time cost (nearly 1 second for each captured image). We instead implemented this conversion on a mobile GPU by means of off-screen rendering technology, which enables rapid processing or rendering of data in an off-screen buffer with the help of the parallel computing capability of a GPU. We implemented the conversion algorithm in OpenGL Shading Language (GLSL) using OpenGL ES v2.0, and we found that our optimized method requires only approximately 15 ms to convert a YUV image into an RGB image on a mobile GPU, which represents a significant reduction in time cost.

### 5.2. Application and Validation

#### 5.2.1. Data Acquisition

**Figure 9:**
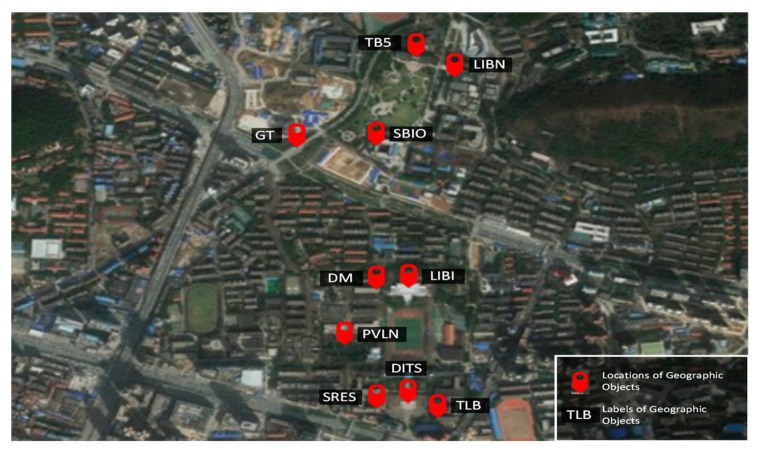
The distribution of the selected geographic objects on the Wuhan University campus. Their locations and labels (actually, assigned codes) are marked on a background image obtained from ESRI ArcGIS Earth v1.5.

We tested the prototype system on the Wuhan University campus to evaluate the method and validate its results. We selected 10 representative geographic objects on the campus, including buildings, famous statues, and pavilions. Some of the selected objects are located very close to each other and often appear together in the same image, thereby allowing us to evaluate the method’s multiple-object detection performance. Some of the objects also share very similar visual properties, allowing us to evaluate the ability to further precisely distinguish them based on their spatial relationships with the help of the built-in GPS receiver, IMU and magnetometer. Moreover, most of these geographic objects are of various sizes, appearances and colours, and various outdoor environmental conditions were encountered during the test, allowing us to thoroughly test the robustness of the method. The distribution of the selected geographic objects is shown in Figure 9.

A corresponding database was established to store geospatial information on these geographic objects. The current geospatial information database stores ID, name (assigned code), category (e.g., building), area (estimated area of the geographic object), height (maximum height of the geographic object), longitude (east longitude), latitude (north latitude) and text introduction of the geographic object. Additional types of information could easily be added by extending the table entries of the database. The structural details of the geospatial information database are shown in Table 2.

To train the proposed lightweight SSD for vision-based detection, we acquired an enormous number of photographs of the geographic objects. Each geographic object corresponds to at least 200 images, and each image contains at least one of the geographic objects, sometimes multiple. We captured approximately 2000 photographs at multiple scales (mainly from different distances) and from multiple perspectives (from different directions) as well as under many different conditions to assist the detector in learning the essential features of the geographic objects. The objective was to allow these geographic objects to be detected in images at any possible size, from various possible view directions and under many possible conditions by a successfully trained detection algorithm.

#### 5.2.2. Preprocessing and Training

**Figure 10:**
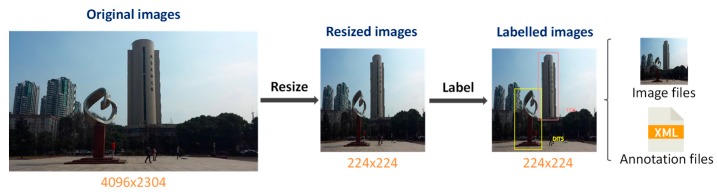
The entire process of image labelling. First, the original images are resized to dimensions of 224 × 224 pixels. Then, all of the geographic objects in the images are manually labelled with classifications and bounding box coordinates. Finally, this information is stored in XML files, and all of the resized images and XML annotation files are organized in the VOC2007 format.

To train the proposed lightweight SSD, images alone are not sufficient. Annotations specifying classifications and bounding box coordinates for each geographic object in the images are needed. We produced a detection dataset in the VOC2007 format for the selected geographic objects (the entire process is illustrated in Figure 10). First, we resized all images to dimensions of 224 × 224 pixels, and then, we manually created annotations by labelling classifications and bounding box coordinates for all of the geographic objects in the images. Those annotations are organized in accordance with the VOC2007 format requirements and are stored in XML files together with all of the resized images in the VOC2007 format. We designated 1000 of the annotated images as the training set, another 500 as the validation set, and the rest as the test set. The training and validation sets were both used to train the detector, and the test set was used to calculate the mAP to evaluate its performance.

**Figure 11:**
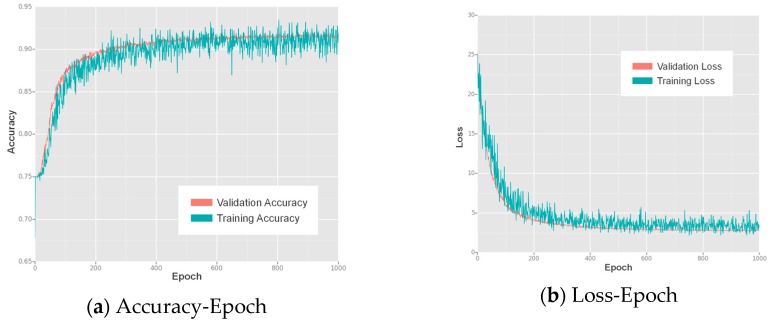
The accuracy and loss values for each epoch of training of the proposed lightweight SSD on the geographic object detection dataset: (**a**) the accuracy-epoch curves; (**b**) the loss-epoch curves.

We trained the lightweight SSD on a desktop PC with an NVIDIA GTX 1060 GPU (with 6 GB of video memory) and an Intel^®^ Core™ i7-6700K CPU @ 4.00 GHz x 8 (with 8 GB of memory). The detector was implemented and trained using MXNet v0.9.0, which supports CUDA, a parallel computing platform for General-Purpose computing on Graphical Processing Units (GPGPU). This hardware and these platforms significantly reduced the time cost for training. We trained the approach for 1000 epochs using the same training configuration that was used in the preliminary network performance evaluation, and the resulting accuracy-epoch and loss-epoch curves are presented in Figure 11. This figure shows that convergence was reached at approximately the 800th epoch, after which the accuracy-epoch and loss-epoch curves remained stable.

#### 5.2.3. Results and Validation

We evaluated the trained lightweight SSD on the test set of the geographic object detection dataset by calculating AP and mAP values. None of the images in the test set was used in the training stage; therefore, this evaluation provides a fair estimate of the actual performance of the proposed detection method. The results are shown in Table 3. Our approach achieves a mAP value of 0.97, which indicates very high vision-based detection accuracy for the selected geographic objects.

Subsequently, we returned to the campus to test the prototype system using the mobile outdoor AR method. The mobile device we used was an Android mobile phone with a Qualcomm Snapdragon 821 mobile CPU, an Adreno 530 mobile GPU, 6 GB of memory, a 16-megapixel Sony IMX 298 sensor, a 5.5-inch Optic AMOLED capacitive touchscreen, a built-in GPS receiver and several inertial/magnetic sensors, including a magnetometer, an accelerometer, and a gyroscope.

**Figure 12:**
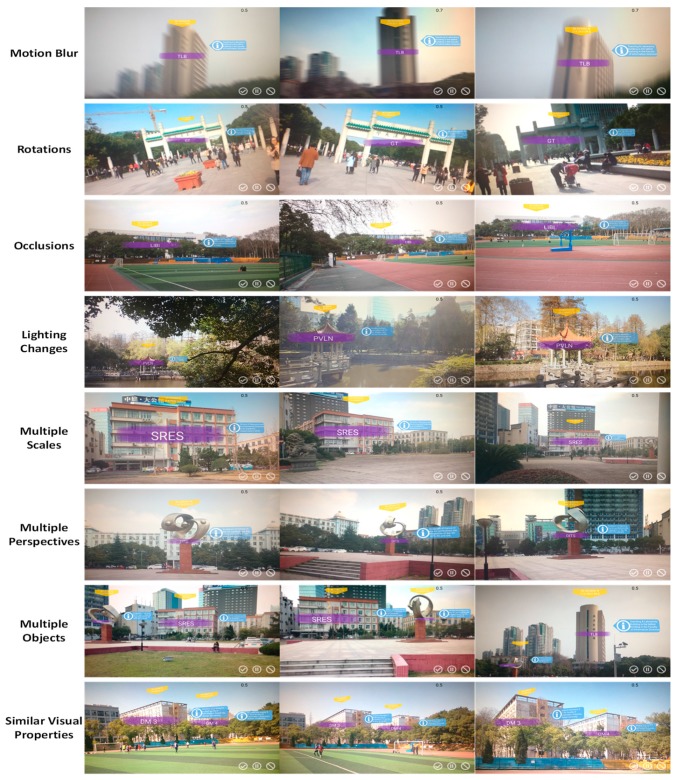
All of the results of the spot tests are shown, covering various conditions, including motion blur (in different directions), rotations, occlusions (by trees and a basketball stand), lighting changes, multiple scales (from different distances), multiple perspectives (from different directions), multiple geographic objects and examples of distinguishing between geographic objects with similar visual properties. During the tests, the geographic objects were simply enhanced with virtual objects indicating their names (purple), GPS locations (yellow) and brief information (blue).

We tested the overall performance under many challenging uncontrolled outdoor conditions, including motion blur, rotation, occlusion, lighting changes, multiple scales, multiple perspectives and multiple objects, and we also tested the performance in distinguishing geographic objects with similar visual properties. All of the test results are presented in Figure 12.

**Figure 13:**
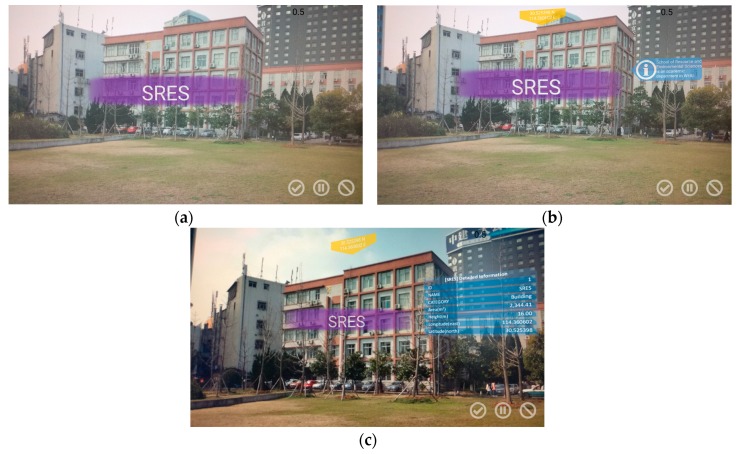
The interactions implemented in the prototype system: (**a**) the system first detects the geographic object labelled SRES and then enhances it with a virtual label; (**b**) after the user touches the label reading “SRES” on the screen, several virtual objects are superimposed on the detected object, displaying some concise geospatial information; (**c**) after the user touches the brief information label (blue), more specific geospatial information is shown.

The first four rows in Figure 12 show that our method can cope with intense motion blur, rotation, occlusion and various lighting conditions. The fifth and sixth rows show that our method can precisely detect the selected geographic objects regardless of which side is facing the visual sensor or at what distance the object lies from the visual sensor, thereby confirming its robust detection performance from multiple perspectives and at multiple scales. The seventh row shows that our method is capable of detecting multiple objects. In the last row, two geographic objects, DM 3 and DM 4 (two dormitories with similar visual properties, which both belong to the “DM” class in the geographic object detection dataset but for which information is separately stored in the geospatial information database), are correctly distinguished, and the corresponding virtual objects superimposed on the screen are displayed with the proper locations and overlay order. The interaction method was also tested; several example screenshots illustrating this process are shown in Figure 13.

In the spot tests, all of the geographic objects were correctly detected, and the system always exhibited high-accuracy registration as well as stable AR geovisualization and interaction. Finally, the average time cost of our method, divided into several basic stages, when running on a mobile phone is reported in Table 4.

### 5.3. Discussion

The experimental results show that our mobile outdoor AR method achieves excellent performance in detecting and enhancing geographic objects in uncontrolled outdoor environments and enriches the user experience. Our method achieves a high detection success rate and accuracy for several reasons. On the one hand, the detection approach we use, the proposed lightweight SSD, is a vision-based deep learning approach, which can learn essential and robust features from image data to achieve highly accurate detection performance that is superior to that of many traditional approaches based on manually crafted features. On the other hand, when constructing the geographic object detection dataset, we used images that captured the selected geographic objects from all perspectives and scales and under many conditions to the greatest possible extent. Doing so facilitated the ability of the lightweight SSD to learn essential and robust features, thereby improving the detection success rate and accuracy. Moreover, the integration of the vision-based detection results with the corresponding spatial relationships with the help of the mobile or embedded device’s built-in GPS receiver, IMU and magnetometer significantly reduces detection errors and enables precise distinction between geographic objects with similar visual properties, which is quite difficult for a purely vision-based detection algorithm to achieve.

Notably, although the lightweight SSD approach achieves a mAP value of 97% on our geographic object detection dataset, its mAP value on the standard VOC2007 test dataset is only 53.7%. The key reason for this enormous difference is that the types of objects in the VOC2007 dataset are abstract, such as “car” and “bottle”, and the purpose of the VOC2007 dataset is to train an algorithm that can detect, for example, all kinds of cars and all kinds of bottles; consequently, it is generally a difficult task to achieve a very high mAP on this dataset. By contrast, the objects included in our geographic object detection dataset are quite specific, and specific objects are much easier to train a detector to detect; consequently, our trained detector can achieve state-of-the-art detection performance and a high mAP.

In addition, the detection and AR geovisualization results of our system are stable. This is because the two essential components of the detection task—classification and localization—are both vision-based and are therefore less susceptible to interference from various outdoor variables, such as electromagnetic fluctuations, and thus more stable than methods based purely on inertial/magnetic sensors, which suffer from drift and distortion. Our AR geovisualization is based on stable detection results combined with spatial relationships, and consequently, the final visualization results satisfy visual expectations.

Furthermore, the weight file of the trained lightweight SSD is much smaller (17.8 MB for one model) than those of most detection frameworks and is thus compatible with the limited storage space of various mobile or embedded devices. With the application of compression technologies, such as deep compression [75], the model size could be further reduced. In addition, the entire AR method is independent of the network; all computing tasks are performed offline by the user’s mobile or embedded device, thereby eliminating the effects of network latency and making the method very flexible and robust for application in challenging uncontrolled outdoor environments with poor signal conditions.

The method also has several limitations. First, the detection approach we adopt in this method is a vision-based approach; consequently, it cannot handle very poor lighting conditions (e.g., night). Second, although our method can detect geographic objects at multiple scales from different distances, it still may fail when the distance between the visual sensor and a geographic object is excessively long or short. When the object is too near, the appearance of the geographic object may be too large to be completely captured by the visual sensor, and thus, the incomplete object in the image may fail to be detected. When the object is too far, the appearance of the geographic object in the image may be too small for successful detection. Therefore, although our lightweight SSD framework is able to detect geographic objects at multiple scales in most cases, it is still necessary to avoid extreme distances between the visual sensor and the geographic objects of interest when using this method. Third, the overall method does not run very fast on a mobile CPU. We simplified the detection approach and optimized the image capture process, and as a result, we achieved near-real-time performance with a rate of approximately 2 FPS; this is faster than many server-based methods, which all suffer from network latency, but it is still not very fast. The most time-consuming step of the method is the lightweight SSD detection; further simplifying the lightweight SSD architecture would reduce the time cost but might also decrease the detection accuracy, leading to a trade-off between speed and accuracy. However, it should be noted that the mobile CPU we used in the test was not the most up-to-date model available; therefore, it is expected that with a higher-performance CPU, the time cost will be further reduced, allowing the method to run at a higher speed. Fourth, the spatial relationships we use such as distances, directions, and orders are effective but still not sufficient for the refinement. For example, our method lacks the application of scene segmentation and the understanding of the spatial layout of outdoor environments, thus it may fail to eliminate some unreasonable results (e.g., it may think that a building floating in the air is reasonable); Also, the spatial relationships we infer rely heavily on the GPS receiver and other sensors, making them vulnerable to the deterioration in the GPS precision and drift and distortion in the output of sensors; Besides, the location of a geographic object is represented only by a pair of GPS coordinate in our method, therefore it will be very challenging to construct correct spatial relationships when the geographic objects with complex structures entangles together.

## 6. Conclusions and Future Work

Traditional forms of geovisualization, such as paper maps and digital maps, are limited by their insufficiently intuitive use and generally provide limited modes of interaction. Outdoor AR is, in principle, a suitable way to visualize geographic data and information by supplementing the real world with virtual objects that users can easily understand and interact with. However, many traditional AR methods have several drawbacks when applied in various outdoor environments: fiducial-marker-based methods are inappropriate for use in uncontrolled environments, whereas sensor-based methods can be overly sensitive to certain variables in outdoor environments, such as magnetic fields, which can easily cause errors and even failures.

In this paper, we proposed a robust, markerless and near-real-time mobile outdoor AR method for geovisualization. We adopted SSD, a vision-based deep learning object detection approach, for the detection of geographic objects based on their natural features under various outdoor conditions. To reduce the computational burden and weight of this approach for mobile outdoor AR, we modified its original structure to obtain lightweight SSD, a energy-efficient, less computationally expensive but still powerful approach. To facilitate registration, we combined the vision-based detection results of the proposed lightweight SSD with the corresponding spatial relationships between objects in the real world with the help of the host device’s built-in GPS receiver, IMU and magnetometer, thereby significantly reducing detection errors and achieving the ability to correctly distinguish between similar geographic objects. Then, we designed and implemented methods of AR geovisualization and interaction based on virtual objects generated from geospatial information and the recognition of touch gestures on mobile or embedded devices. Because of the poor signal conditions found in many challenging outdoor environments, we chose to take full advantage of mobile computing capabilities by means of a performance optimization scheme that allows all computational tasks to be executed on the user’s mobile or embedded device, thereby eliminating any dependence on the network and eradicating latency. Finally, we developed a prototype system on the Android platform and tested it on the Wuhan University campus using several representative geographic objects to evaluate the method and validate its results.

The findings demonstrate that our method has a high detection success rate and accuracy, produces stable AR geovisualization results, and is lightweight and flexible by virtue of its small model size and network independence. All of these features make it very suitable for use in uncontrolled outdoor environments. The system benefits from the combination of geovisualization with mobile outdoor visual-IMU-magnetometer AR, and it also reflects the potential, high accuracy and robustness of deep-learning-based approaches for geographic object detection. Furthermore, this work offers a new way to visualize geographic data and information and to interact with such information in the real world through mobile outdoor AR, which enriches the user experience and is expected to be beneficial for various applications related to geospatial information.

Our future research will focus on modifying the architecture of the detection approach to make it lighter, faster and more accurate; Using layout estimation and scene segmentation to generate more robust and powerful spatial relationships for understanding the outdoor environment and refining detection results; integrating a high-speed object tracking algorithm into the system to further reduce the time cost; and incorporating a pose estimation algorithm to precisely estimate the orientations of geographic objects.

## Figures and Tables

**Table 1:** Comparison between original SSD, Fast YOLO and lightweight SSD.

Framework	mAP	Model Size	FPS on GPU (PC) ^1^	FPS on CPU (PC) ^2^	FPS on CPU (Mobile) ^3^
Original SSD	74.3%	104.3 MB	11.6	1.5	0.2
Fast YOLO	52.7%	64.7 MB	30.1	5.9	-
Lightweight SSD	53.7%	17.8 MB	66.7	9.1	2.0

^1^ The GPU we used in the PC was an NVIDIA GeForce GTX 1060 with 6 GB of video memory.

^2^ The CPU we used in the PC was an Intel^®^ Core™ i7-6700K CPU @ 4.00 GHz × 8 with 8 GB of memory.

^3^ The CPU we used on the mobile phone was a Qualcomm Snapdragon 821 with 6 GB of memory.

**Table 2:** Structural details of the geospatial information database.

Item	Data Type	Example ^1^
ID	Integer	1
Name	Text	SRES
Category	Text	Building
Area (m^2^)	Real	2,344.41
Height (m)	Real	16.00
Longitude (East)	Real	114.360602
Latitude (North)	Real	30.525398
Introduction	Text	The School of Resources and Environmental Sciences is an academic department at WHU.

^1^ The example data and information are just for reference.

**Table 3:** AP and mAP values achieved on the test set by the trained lightweight SSD for the selected geographic objects.

	SRES	DITS	TLB	PVLN	LIBI	DM	GT	SBIO	LIBN	TB5
AP	0.97	0.95	0.96	0.95	0.98	0.89	0.99	0.99	0.99	0.99
mAP	0.97									

**Table 4:** The average time cost of the method divided into different stages when running on a mobile phone ^1^.

Basic Steps	Average Time Cost (ms)
Image Capture	2
Image Resizing	6
YUV-to-RGB Conversion	15
Lightweight SSD Detection	520
Spatial Relationships Combination	15
AR Visualization	2
Total Time Cost (ms)	560

^1^ The CPU of the mobile phone we used is a Qualcomm Snapdragon 821 mobile CPU. On other mobile phones with higher-performance CPUs, the time cost is expected to be lower.

## References

[1] MacEachren A.M., Gahegan M., Pike W., Brewer I., Cai G., Lengerich E., Hardistry F. (2004). Geovisualization for knowledge construction and decision support. IEEE Comput. Graph. Appl..

[2] Grünfeld K. (2005). Integrating spatio-temporal information in environmental monitoring data-a visualization approach applied to moss data. Sci. Total Environ..

[3] Andrienko G., Andrienko N., Jankowski P., Keim D., Kraak M.J., MacEachren A., Wrobel S. (2007). Geovisual analytics for spatial decision support: Setting the research agenda. Int. J. Geogr. Inf. Sci..

[4] Sagl G., Loidl M., Beinat E. (2012). A visual analytics approach for extracting spatio-temporal urban mobility information from mobile network traffic. ISPRS Int. J. Geo Inf..

[5] Lu M., Chen M., Wang X., Min J., Liu A. (2017). A spatial lattice model applied for meteorological visualization and analysis. ISPRS Int. J. Geo Inf..

[6] Watters M.S. (2006). Geovisualization: An example from the catholme ceremonial complex. Archaeol. Prospect..

[7] Li R. (2017). Effects of visual variables on the perception of distance in off-screen landmarks: Size, color value, and crispness. Progress in Location-Based Services 2016.

[8] MacEachren A.M. (1994). Visualization in modern cartography: Setting the agenda. Vis. Mod. Cartogr..

[9] Portalés C., Lerma J.L., Navarro S. (2010). Augmented reality and photogrammetry: A synergy to visualize physical and virtual city environments. ISPRS J. Photogramm. Remote. Sens..

[10] Hedley N.R., Billinghurst M., Postner L., May R., Kato H. (2002). Explorations in the use of augmented reality for geographic visualization. Presence Teleoper. Virtual Environ..

[11] Tayara H., Ham W., Chong K.T. (2016). A real-time marker-based visual sensor based on a FPGA and a soft core processor. Sensors.

[12] Kato H., Billinghurst M. Marker Tracking and Hmd Calibration for a Video-Based Augmented Reality Conferencing System. Proceedings of the 2nd IEEE and ACM International Workshop on Augmented Reality, 1999 (IWAR’99).

[13] Azuma R., Baillot Y., Behringer R., Feiner S., Julier S., MacIntyre B. (2001). Recent advances in augmented reality. IEEE Comput. Graph. Appl..

[14] Chen J., Cao R., Wang Y. (2015). Sensor-aware recognition and tracking for wide-area augmented reality on mobile phones. Sensors.

[15] Tsai M.K., Lee Y.C., Lu C.H., Chen M.H., Chou T.Y., Yau N.J. (2012). Integrating geographical information and augmented reality techniques for mobile escape guidelines on nuclear accident sites. J. Environ. Radioact..

[16] Ghadirian P., Bishop I.D. (2008). Integration of augmented reality and GIS: A new approach to realistic landscape visualisation. Landsc. Urban Plan..

[17] Schall G., Wagner D., Reitmayr G., Taichmann E., Wieser M., Schmalstieg D., Hofmann-Wellenhof B. Global Pose Estimation Using Multi-Sensor Fusion for Outdoor Augmented Reality. Proceedings of the 8th IEEE International Symposium on Mixed and Augmented Reality.

[18] Duan L., Guan T., Yang B. (2009). Registration combining wide and narrow baseline feature tracking techniques for markerless ar systems. Sensors.

[19] Guan T., Duan L., Chen Y., Yu J. (2010). Fast scene recognition and camera relocalisation for wide area augmented reality systems. Sensors.

[20] Lowe D.G. (2004). Distinctive image features from scale-invariant keypoints. Int. J. Comput. Vis..

[21] Bay H., Ess A., Tuytelaars T., Van Gool L. (2008). Speeded-up robust features (surf). Comput. Vis. Image Underst..

[22] Dalal N., Triggs B. Histograms of Oriented Gradients for Human Detection. Proceedings of the 2005 IEEE Computer Society Conference on Computer Society Conference on Computer Vision and Pattern Recognition.

[23] Viola P., Jones M. Rapid Object Detection Using a Boosted Cascade of Simple Features. Proceedings of the 2001 IEEE Computer Society Conference on Computer Vision and Pattern Recognition.

[24] Hagbi N., Bergig O., El-Sana J., Billinghurst M. (2011). Shape recognition and pose estimation for mobile augmented reality. IEEE Trans. Vis. Comput. Graph..

[25] Huang W., Sun M., Li S. (2016). A 3D GIS-based interactive registration mechanism for outdoor augmented reality system. Expert Syst. Appl..

[26] Feiner S., MacIntyre B., Hollerer T., Webster A. A Touring Machine: Prototyping 3D Mobile Augmented Reality Systems for Exploring the Urban Environment. Proceedings of the First International Symposium on Wearable Computers, Digest of Papers.

[27] Foxlin E. (2005). Pedestrian tracking with shoe-mounted inertial sensors. IEEE Comput. Graph. Appl..

[28] Yohan S.J., Julier S., Baillot Y., Lanzagorta M., Brown D., Rosenblum L. Bars: Battlefield Augmented Reality System. Proceedings of the NATO Symposium on Information Processing Techniques for Military Systems.

[29] Piekarski W., Thomas B.H. Tinmith-Metro: New Outdoor Techniques for Creating City Models with An Augmented Reality Wearable Computer. Proceedings of the Fifth International Symposium on Wearable Computers.

[30] Azuma R., Hoff B., Neely H., Sarfaty R. A Motion-Stabilized Outdoor Augmented Reality System. Proceedings of the IEEE Virtual Reality.

[31] Lee S., Suh J., Park H.-D. (2015). Boreholear: A mobile tablet application for effective borehole database visualization using an augmented reality technology. Comput. Geosci..

[32] Behringer R. Registration for Outdoor Augmented Reality Applications Using Computer Vision Techniques and Hybrid Sensors. Proceedings of the IEEE Virtual Reality.

[33] You S., Neumann U., Azuma R. Hybrid Inertial and Vision Tracking for Augmented Reality Registration. Proceedings of the IEEE Virtual Reality.

[34] Jiang B., Neumann U., You S. A Robust Hybrid Tracking System for Outdoor Augmented Reality. Proceedings of the IEEE Virtual Reality.

[35] Azuma R., Neely H., Daily M., Leonard J. Performance Analysis oF an Outdoor Augmented Reality Tracking System That Relies Upon A Few Mobile Beacons. Proceedings of the 5th IEEE and ACM International Symposium on Mixed and Augmented Reality.

[36] Reitmayr G., Drummond T. Going Out: Robust Model-Based Tracking for Outdoor Augmented Reality. Proceedings of the 5th IEEE and ACM International Symposium on Mixed and Augmented Reality.

[37] Behringer R., Park J., Sundareswaran V. Model-Based Visual Tracking for Outdoor Augmented Reality Applications. Proceedings of the International Symposium on Mixed and Augmented Reality.

[38] Behzadan A.H., Kamat V.R. (2007). Georeferenced registration of construction graphics in mobile outdoor augmented reality. J. Comput. Civ. Eng..

[39] Chen D.M., Tsai S.S., Vedantham R., Grzeszczuk R., Girod B. Streaming Mobile Augmented Reality on Mobile Phones. Proceedings of the 8th International Symposium on Mixed and Augmented Reality.

[40] Skrypnyk I., Lowe D.G. Scene Modelling, Recognition and Tracking with Invariant Image Features. Proceedings of the Third IEEE and ACM International Symposium on Mixed and Augmented Reality, 2004.

[41] Takacs G., Chandrasekhar V., Gelfand N., Xiong Y., Chen W.C., Bismpigiannis T., Grzeszczuk R., Pulli K., Girod B. Outdoors Augmented Reality on Mobile Phone Using Loxel-Based Visual Feature Organization. Proceedings of the 1st ACM International Conference on Multimedia Information Retrieval.

[42] Wagner D., Reitmayr G., Mulloni A., Drummond T., Schmalstieg D. (2010). Real-time detection and tracking for augmented reality on mobile phones. IEEE Trans. Vis. Comput. Graph..

[43] Barandiaran I., Paloc C., Graña M. (2010). Real-time optical markerless tracking for augmented reality applications. J. Real Time Image Process..

[44] Jain P., Manweiler J., Roy Choudhury R. Overlay: Practical Mobile Augmented Reality. Proceedings of the 13th Annual International Conference on Mobile Systems Applications and Services.

[45] Shahrokni A., Vacchetti L., Lepetit V., Fua P. Polyhedral Object Detection and Pose Estimation for Augmented Reality Applications. Proceedings of the Computer Animation 2002.

[46] Chen X., Kundu K., Zhang Z., Ma H., Fidler S., Urtasun R. Monocular 3D Object Detection for Autonomous Driving. Proceedings of the IEEE Conference on Computer Vision and Pattern Recognition.

[47] Han J., Zhang D., Cheng G., Guo L., Ren J. (2015). Object detection in optical remote sensing images based on weakly supervised learning and high-level feature learning. IEEE Trans. Geosci. Remote Sens..

[48] Tang T., Zhou S., Deng Z., Zou H., Lei L. (2017). Vehicle detection in aerial images based on region convolutional neural networks and hard negative example mining. Sensors.

[49] Takeki A., Trinh T.T., Yoshihashi R., Kawakami R., Iida M., Naemura T. (2016). Combining deep features for object detection at various scales: Finding small birds in landscape images. IPSJ Trans. Comput. Vis. Appl..

[50] Solaiman B., Burdsall B., Roux C. Hough Transform and Uncertainty Handling. Application to Circular Object Detection in Ultrasound Medical Images. Proceedings of the 1998 International Conference on Image Processing.

[51] Deng J., Dong W., Socher R., Li L.J., Li K., Fei-Fei L. Imagenet: A Large-Scale Hierarchical Image Database. Proceedings of the CVPR 2009. IEEE Conference on Computer Vision and Pattern Recognition.

[52] Everingham M., Van Gool L., Williams C.K., Winn J., Zisserman A. (2010). The pascal visual object classes (voc) challenge. Int. J. Comput. Vis..

[53] Lin T.-Y., Maire M., Belongie S., Hays J., Perona P., Ramanan D., Dollár P., Zitnick C.L. Microsoft Coco: Common Objects in Context. Proceedings of the European conference on computer vision.

[54] LeCun Y., Bengio Y., Hinton G. (2015). Deep learning. Nature.

[55] Krizhevsky A., Sutskever I., Hinton G.E. Imagenet Classification with Deep Convolutional Neural Networks, Advances in Neural Information Processing Systems. https://papers.nips.cc/paper/4824-imagenet-classification-with-deep-convolutional-neural-networks.pdf.

[56] Sánchez J., Perronnin F. High-Dimensional Signature Compression for Large-Scale Image Classification. Proceedings of the 2011 IEEE Conference on Computer Vision and Pattern Recognition (CVPR).

[57] Sermanet P., Eigen D., Zhang X., Mathieu M., Fergus R., LeCun Y. Overfeat: Integrated Recognition, Localization and Detection Using Convolutional Networks. https://arxiv.org/pdf/1312.6229.pdf.

[58] Hosang J., Benenson R., Dollár P., Schiele B. (2016). What makes for effective detection proposals?. IEEE Trans. Pattern Anal. Mach. Intel..

[59] Van de Sande K.E., Uijlings J.R., Gevers T., Smeulders A.W. Segmentation as Selective Search for Object Recognition. Proceedings of the 2011 IEEE International Conference on Computer Vision (ICCV).

[60] Alexe B., Deselaers T., Ferrari V. What Is An Object?. Proceedings of the 2010 IEEE Conference on Computer Vision and Pattern Recognition.

[61] Girshick R., Donahue J., Darrell T., Malik J. Rich Feature Hierarchies for Accurate Object Detection and Semantic Segmentation. Proceedings of the IEEE conference on computer vision and pattern recognition.

[62] He K., Zhang X., Ren S., Sun J. (2015). Spatial pyramid pooling in deep convolutional networks for visual recognition. IEEE Trans. Pattern Anal. Mach. Intell..

[63] Girshick R. Fast R-Cnn. Proceedings of the IEEE International Conference on Computer Vision.

[64] Ren S., He K., Girshick R., Sun J. (2017). Faster R-CNN: Towards real-time object detection with region proposal networks. IEEE Trans. Pattern Anal. Mach. Intell..

[65] Redmon J., Divvala S., Girshick R., Farhadi A. You Only Look Once: Unified, Real-Time Object Detection. Proceedings of the IEEE Conference on Computer Vision and Pattern Recognition.

[66] Liu W., Anguelov D., Erhan D., Szegedy C., Reed S., Fu C.-Y., Berg A.C. Ssd: Single Shot Multibox Detector. Proceedings of the European Conference on Computer Vision.

[67] Simonyan K., Zisserman A. Very Deep Convolutional Networks for Large-Scale Image Recognition. https://arxiv.org/pdf/1409.1556.pdf.

[68] Iandola F.N., Han S., Moskewicz M.W., Ashraf K., Dally W.J., Keutzer K. Squeezenet: Alexnet-Level Accuracy with 50 × Fewer Parameters and <0.5 mb Model Size. https://arxiv.org/pdf/1602.07360.pdf.

[69] Schneiderman H., Kanade T. Probabilistic Modeling of Local Appearance and Spatial Relationships for Object Recognition. Proceedings of the Computer Society Conference on Computer Vision and Pattern Recognition.

[70] Choi W., Chao Y.-W., Pantofaru C., Savarese S. Understanding Indoor Scenes Using 3D Geometric Phrases. Proceedings of the IEEE Conference on Computer Vision and Pattern Recognition.

[71] Li J., Meger D., Dudek G. Learning to Generalize 3D Spatial Relationships. Proceedings of the 2016 IEEE International Conference on Robotics and Automation (ICRA).

[72] Hoiem D., Efros A.A., Hebert M. (2008). Putting objects in perspective. Int. J. Comput. Vis..

[73] Sun M., Bao Y., Savarese S. (2010). Object detection with geometrical context feedback loop. BMVC.

[74] Chen T., Li M., Li Y., Lin M., Wang N., Wang M., Xiao T., Xu B., Zhang C., Zhang Z. Mxnet: A Flexible and Efficient Machine Learning Library for Heterogeneous Distributed Systems. https://arxiv.org/pdf/1512.01274.pdf.

[75] Han S., Mao H., Dally W.J. Deep Compression: Compressing Deep Neural Networks with Pruning, Trained Quantization and Huffman Coding. https://arxiv.org/pdf/1510.00149.pdf.

